# A Brief History and Future Prospects of CEST MRI in Clinical Non-Brain Tumor Imaging

**DOI:** 10.3390/ijms222111559

**Published:** 2021-10-26

**Authors:** Tianxin Gao, Chuyue Zou, Yifan Li, Zhenqi Jiang, Xiaoying Tang, Xiaolei Song

**Affiliations:** 1 School of Life Science, Institute of Engineering Medicine, Beijing Institute of Technology, Beijing 100081, China; gtx@bit.edu.cn (T.G.); zou15211137@163.com (C.Z.); 7520200073@bit.edu.cn (Z.J.); 2 Center for Biomedical Imaging Research, School of Medicine, Tsinghua University, Beijing 100084, China; yifan.li@pku.edu.cn

**Keywords:** chemical exchange saturation transfer, body tumor, clinical scanner, amide proton transfer

## Abstract

Chemical exchange saturation transfer (CEST) MRI is a promising molecular imaging tool which allows the specific detection of metabolites that contain exchangeable amide, amine, and hydroxyl protons. Decades of development have progressed CEST imaging from an initial concept to a clinical imaging tool that is used to assess tumor metabolism. The first translation efforts involved brain imaging, but this has now progressed to imaging other body tissues. In this review, we summarize studies using CEST MRI to image a range of tumor types, including breast cancer, pelvic tumors, digestive tumors, and lung cancer. Approximately two thirds of the published studies involved breast or pelvic tumors which are sites that are less affected by body motion. Most studies conclude that CEST shows good potential for the differentiation of malignant from benign lesions with a number of reports now extending to compare different histological classifications along with the effects of anti-cancer treatments. Despite CEST being a unique ‘label-free’ approach with a higher sensitivity than MR spectroscopy, there are still some obstacles for implementing its clinical use. Future research is now focused on overcoming these challenges. Vigorous ongoing development and further clinical trials are expected to see CEST technology become more widely implemented as a mainstream imaging technology.

## 1. Introduction

Magnetization transfer (MT) technology in magnetic resonance imaging (MRI), referring to the transfer of longitudinal magnetization between two proton groups, was first proposed by Wolff and Balaban et al. in 1989 [1]. Conventional MT is the transfer of magnetization between water and semisolid macromolecules. In 1998, Guivel-Scharen observed the asymmetry of the Z-spectrum near the resonance frequency of water when studying the MT phenomenon of small molecule solutions [2]. Later, in the year 2000, by combining magnetization transfer and chemical exchange, Wolff and Balaban first obtained the MR contrast images of several small molecules and named this novel molecular imaging technique chemical exchange saturation transfer (CEST) [3].

This imaging technology has attracted a number of preclinical and clinical research studies [4,5], becoming a promising molecular imaging tool that is available in the clinic [6,7]. Particularly, CEST imaging has been explored in assessing tumor metabolism, pH microenvironment, and histological types [4,5,6]. Like other MR techniques, CEST has been intensively investigated for characterizing brain tumors, with several dedicated reviews [6,8]. CEST has also been widely studied in non-brain tumors, especially in recent years with the progress in CEST acquisition sequences and post-processing methods. Compared with brain tumor imaging, CEST imaging of body tumors faces several common technical challenges including fat interference, motion artifacts, the B_0_/B_1_ inhomogeneity, and the power restrictions that are required for a larger field of view than the brain. Compared with the brain, body imaging also has other unique properties, including the absence of the blood-brain-barrier and more heterogeneous tissue composition. To address the current status and future prospects of body CEST imaging, this review provides a survey of the application of CEST for imaging various tumors throughout the body, in particular methods that are performed as part of clinical imaging applications.

**Figure 1:**
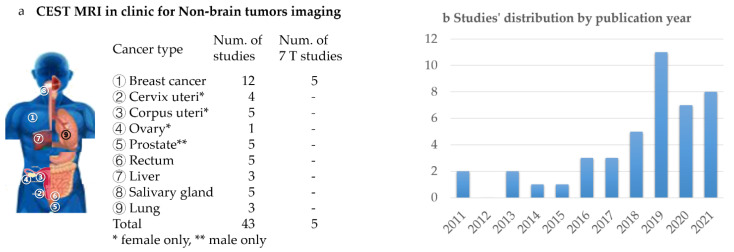
Scope of the review, and an overview summary for the studies that were included. (**a**) Cancer types and the corresponding num. of studies included in the review, with 5 breast cancer studies performed on 7T MR scanners, and the rest performed on 3T scanners; (**b**) Studies’ distribution by publication year.

The literature search was performed through the electronic databases of Web of Science Core Collection for original studies that were published in English up to 30 September 2021. The keywords of the included studies covered three domains: 1: “chemical exchange saturation transfer” or “amide proton transfer”, 2: “tumor” or “cancer”, 3: “clinical” or “patient”. The exclusion criterion were the studies that related to “brain” or “glioma”. The complete search strategy was ((TS = (“Chemical Exchange Saturation Transfer” *)) OR (TS = (“Amide Proton Transfer”*))) AND ((TS = (tumor*)) OR (TS = (cancer*))) AND (TS = (clinical*) OR (TS = (patient*))) NOT (TS = (brain OR glioma*)). Of the 92 studies that were found, 65 were non-review articles, in which 44 focused on the clinical usage of CEST with human subjects. The tumor types that were reviewed here and the relevant references that were identified are listed in Figure 1.

## 2. Principle of CEST

### 2.1. Basic Theory

**Figure 2:**
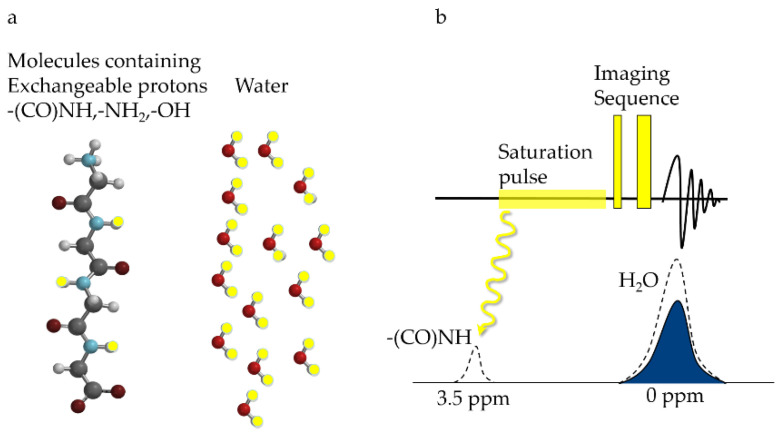
Illustration of CEST principle. (**a**) signal sources of CEST, which includes solute molecules containing exchangeable protons (highlighted in yellow), and the surrounding water; (**b**) the MR pulse sequence for CEST detection, which adds a saturation pulse at the resonance frequency of exchangeable protons (e.g., 3.5 ppm for –(CO)NH), before the conventional water signal readout. The saturation pulse diminishes the signal of the solute protons, which later transfers to water and is amplified through multiple chemical exchanges, causing a reduction in the water signal that could be detected.

CEST relies on the frequency-specific saturation of exchangeable protons on the detection molecules, with the saturated protons later transferred to surrounding water through multiple chemical exchange processes. To mathematically describe the CEST signal, Zhou et al. in 2004 [9] proposed a dual-pool model with exchange items, including a water pool and a solute pool. By selectively applying a radio-frequency (RF) saturation pulse at the resonance frequency of the exchangeable protons in the solute pool, the saturated solute protons transfer to the surrounding water pool through chemical exchange, resulting in decreases in the magnetic resonance signal of water [10]. As shown in Figure 2, the signal will decrease until a dynamic equilibrium of the chemical exchange is reached. By measuring the changes in water molecule signals, information about the solute molecules of interest, as well as the microenvironment, can be indirectly obtained. As the saturation and exchange process continually repeats, the reduction of water molecule signals is much greater than the signal intensity of the solute itself, making the minimal detectable concentrations as low as micromolar levels [11].

To achieve effective saturation transfer, two conditions are necessary. First, the resonant frequency difference between the two exchanging proton pools is greater than the forward (from solute to water) exchange rate ($∆\omega >k_{\mathrm{sw}}$), so that an effective exchange can be achieved. Second, the forward exchange rate is greater than the longitudinal relaxation rate of the protons of the solute pool ($k_{\mathrm{sw}}>R_{1s}$), ensuring sufficient time for the exchange before complete relaxation [12].

Hydrogen protons in different chemical groups have different resonance frequencies due to their chemical environment, the offset of which from the resonance frequency of the hydrogen protons in free water (ω_0_) is an important characteristic, denoted as Δω (which is usually expressed in parts per million (ppm) of ω_0_), so that it keeps constant under different static magnetic fields ($B_{0}$). For example, amide protons resonate at 3.5 ppm from water. The normalized curve of the water signal along with the frequency offsets of the saturation pulses, namely a Z-spectrum, will display a ‘dip’ at Δω, owing to the saturated signal that is transferred from the on-resonance proton groups to the water [13].

### 2.2. CEST Quantification

Compared with the intensity of unsaturated signals, signal reductions at certain frequency offsets derive not only from CEST, but also from the direct saturation (DS) of water, and moreover, from the MT effect of semisolid macromolecules during in vivo imaging. DS is symmetrical with respect to the resonance frequency of water, and the majority of MT is also symmetrical. Thus, the symmetrical effects can be removed by taking the difference between signal intensities at two opposite frequency offsets. This approach describes the idea of asymmetric analysis, a commonly used quantification approach that was proposed by Guivel et al. [2]. The measurement index is expressed as: ${\mathrm{MTR}}_{\mathrm{asym}}=\frac{\left[S\left(-∆\omega \right)-S\left(+∆\omega \right)\right]}{S_{0}}$, where $S_{0}$ refers to the water signal intensity that is obtained when no pre-saturation pulse is applied, $S\left(+∆\omega \right)$ and $S\left(-∆\omega \right)$ refer to the signal intensities that are obtained after applying pre-saturation pulses at + Δω and –Δω, respectively [13,14]. However, MTR_asym_ is unable to separate the CEST signals that are resonating down-field of water (Δω between 1–3.5 ppm), from the nuclear Overhauser effect (NOE) that is resonating up-field of water (Δω between −1.6 to −4 ppm) (see details in 2.3.4).

Another prevailing method of separating pure CEST signals is to subtract the experimental from the reference values that are free from CEST at a certain frequency offset (i.e., $\mathrm{MTR}=Z_{ref}-Z_{exp}$). The latter can be estimated by different algorithms, including multi-pool Lorentzian fitting [15,16], Lorentzian difference (LD) [17], voxel-wise optimization of pseudo Voigt profile (VOPVP) [18], the extrapolated semisolid MT model reference (EMR) approach [19], the three-offset method [20], and the multi-pool Bloch-McConnell fitting [21].

Based on similar estimation approaches of reference Z-spectra, inverse Z-spectrum analysis has been also used in some studies [22], according to which the size of the CEST effect is expressed as ${\mathrm{MTR}}_{Rex}=\frac{1}{Z_{exp}}-\frac{1}{Z_{ref}}$. Furthermore, to eliminate the influence of T_1_ relaxation on the calculation of ${\mathrm{MTR}}_{Rex}$, the apparent exchange-dependent relaxation (AREX) was proposed and denoted as $\mathrm{AREX}\;={\mathrm{MTR}}_{Rex}/T_{1w}$, where the subscript *w* represents free water.

From the magnitude, width, and the frequency offsets of the CEST spectral peaks, as well as the signal dependence on the saturation length and power, information regarding the exchangeable protons on the solutes can be obtained [14]. Specifically, the solute concentrations and the microenvironmental pH could be sensed by means of clever designs and algorithms [23,24].

### 2.3. CEST Effects from Different Proton Groups

CEST effects can be classified into several categories according to the resonance frequencies of the exchangeable protons on the endogenous metabolites that include amide groups (-$\left(\mathrm{CO}\right)\mathrm{NH}$), amine groups (-${\mathrm{NH}}_{2}$), and hydroxyl groups (-OH).

#### 2.3.1. Imaging of Amide Protons

CEST can detect amide protons (-$\left(\mathrm{CO}\right)\mathrm{NH}$), resonating 3.5 ppm from water) on endogenous proteins and peptides, with the underlying phenomenon called amide proton transfer (APT) [25]. The first contrast images that were obtained of proteins and peptides using CEST technology were reported in 2003 by Zhou et al., achieving the detection of in vivo pH changes in an ischemic rat brain [25]. APT imaging was later used to achieve brain tumor imaging in rats, and thereafter in 2008, brain tumor imaging of human patients [26,27]. Compared with the amine and hydroxyl protons, amide protons resonate further to water protons and also exchange slower. Therefore, APT detection is less affected by field inhomogeneity and does not require high saturation B_1_ as it does for the detection of the faster exchanged amines and hydroxyls.

MTR_asym_ (3.5 ppm) is the most widely used metric for APT, which has demonstrated correlations with histological grade in brain tumors and could differentiate tumor recurrence from radiation necrosis [6,8,28]. However, MTR_asym_ (3.5 ppm) includes multiple saturation-transfer effects from amide protons (3.5 ppm), aliphatic protons (−3.5 ppm), and semisolid macromolecules and is, therefore, termed an APT-weighted (APTw) image. Nevertheless, the underlining mechanism of increased APTw signals in malignant tumors remains unclear, but proteomics analyses suggest an association with the abundance of certain metabolic proteins that are found in tumor tissues that are showing abnormal proliferation.

#### 2.3.2. Imaging of Amine Protons

The amine (-${\mathrm{NH}}_{2}$) proton exhibits a peak frequency offset of ~3 ppm from water, but with a faster exchange rate than amide groups [29]. Amine contrast images were obtained either by the CEST technique with a high $B_{1}$ (>2 μT) or by another imaging sequence called spin-lock [29]. The endogenous contrast was assigned to the amine groups on proteins and peptides. The amine signal values (MTR_asym_) at 3 ppm were found to differentiate between two major genotypes of gliomas, namely those that were expressing wild-type and mutant forms of isocitrate dehydrogenase 1 (IDH1). Additionally, the endogenous amine signals were believed to also be derived from the small molecule, glutamate, which is a common excitatory neurotransmitter in the central nervous system and also an important cell metabolite [30]. Cai et al. first used chemical exchange saturation transfer technology to image glutamate in vivo (GluCEST), with the altered glutamate content in lesions validated by MRS [30].

There is also a specific amine proton resonating ~2 ppm from water called guanidine amine. Creatine (Cr) and phosphor creatine (pCr) contain guanidine amine and amine groups, which can be detected by CEST. Potentially CrCEST and pCrCEST can provide assessments of tissue energy metabolism [31]. Using animal models of gliomas with different aggressiveness, CrCEST signals were found to be reduced within the tumor region, with highly aggressive tumors exhibiting more significant signal decreases [4]. The altered creatine concentration was explained by the decreased creatine kinase activity that was associated with increases in the degree of the tumor malignancy [32].

#### 2.3.3. CEST Imaging of Hydroxyl Protons

Exchangeable hydroxyl protons are rich in glycan-containing endogenous molecules, including glycosaminoglycan (GAG) [33], glycogen [34,35], and glycoproteins [36], as well as glucose which is often used as an exogenous contrast agent.

Glycosaminoglycan (GAG), an important component of cartilage tissue, contains one -NH group and three -OH groups in each unit that can be detected by CEST. The GAGCEST technique was developed by Ling et al. [37] for mapping GAG content in vivo. This technique is considered a highly sensitive method for the assessment of GAG levels in cartilage and in intervertebral discs.

Additionally, on the basis that altered glycosylation is a critical hallmark of cancer development, Song et al. [36] applied CEST as a ‘label-free’ cellular imaging method for assessing the different protein glycosylation levels that are expressed in cancers. Ex vivo protein and cell experiments, as well as in vivo animal experiments demonstrated the differentiation of malignant tumors that were expressing under-glycosylated mucin-1 (uMUC1) from uMUC1 negative tumors.

#### 2.3.4. Aliphatic Protons

The NOE was discovered in 1951 by Albert Overhauser [38], which is dipole-dipole coupling that occurs when the spatial distance of two nuclei is smaller than a critical value, manifesting as a change in the NMR signal intensity in one of the two nuclei. For many years, CEST studies focused on metabolites that were featuring positive frequency offsets on the Z-spectra. In 2007, Ling et al. identified a signal drop that was centered at −3.5 ppm on Z-spectra (up-field from the water) and assigned it to NOE [37]. More specifically, the NOE signal derives from magnetization transfer between free water and bound water that is connected with aliphatic chains ((-CH_2_-)_n_) on lipids. At 3 T clinical field strength, NOE could induce an up to 10% water signal drop and show a clear Z-spectra ‘dip’ for the human brain. Therefore, it is considered a promising method for imaging lipid metabolism in various diseases [39].

While in most cases NOE imaging is used to detect lipids, it also has the potential to recognize other molecules. For instance, the glycogen NOE (glycoNOE) signal at around −1 ppm is related to glycogen concentrations [34].

## 3. Technical Issues for Non-Brain Tumor Imaging

To describe the methods and parameters that were employed in the current CEST clinical acquisition and analyses protocols, Table 1 lists the common parameters for all of the studies that were involved in the imaging of various tumor types. Among all of the 43 studies, only 5 studies in breast cancer imaging were investigated at 7 T human scanners, whereas the remaining studies were all performed under 3 T field strength in clinical routine MR scanners. In addition, only 4 studies had a patient population larger than 100, while the rest had a patient number smaller than 100.

The sub-millimeter in-plane resolution can be achieved for a breast cancer study performed at 7 T [40], and a 1–2 mm in-plane resolution has been proven to be feasible for several types of tumor at 3 T scanners. This suggested that the much higher sensitivity of CEST could be achieved over the MR spectroscopy for imaging tumor metabolism. Except for 5 studies that scanned longer, the acquisition time was usually within 7 min. This also reflected the feasibility of CEST for clinical use.

For the quantitation metrics, approximately half of the studies used APTw, i.e., MTRasym (3.5 ppm). This is because APTw is the only commercial CEST imaging protocol that is available on 3 T clinical MR scanners and demonstrated a correlation with the histological grades in brain tumors [6]. Other metrics included Lorentzian fitting (LD), MTRasym at other offsets than 3.5 ppm, and AREX, which were a bit more complicated but allowed better differentiations of signal sources, for example the amide signal from proteins/peptides versus NOE from lipids.

Additionally, body CEST imaging faces several common technical issues, which attracted many efforts in acquisition sequences and analysis methods.

### 3.1. Fat Suppression

Compared with brain imaging, body imaging always needs to consider the interference from fat. A total of three types of sequences were employed in Table 1, which were Dixon-based methods, chemical shift-based methods (SPAIR and SPIR), as well as the use of a frequency-specific excitation pulse for water imaging. Zhang et al. [45] developed CEST-Dixon imaging sequence, allowing for both water-fat separation and $B_{0}$ mapping. For different types of breast cancer tissue, the CEST-Dixon sequence showed homogenous fat removal in water-only images, and also allowed the imaging of hydroxyl protons post B_0_ correction. The Dixon-type sequence was also employed in CEST imaging for rectum tumors and uterus tumors. SPIR or SPAIR are the most commonly used methods for fat suppression, which added a fat-selective inversion pulse and a read signal when fat recovered to zero.

### 3.2. B_0_ and B_1_ Corrections

As a chemical shift-based method, CEST acquisition and analysis are very fragile to B_0_ field inhomogeneity. Local B_0_ shift will cause an inaccurate saturation frequency, artifacts, or image deformation for gradient-echo based imaging sequences, as well as quantification errors. Therefore, CEST usually needs to sweep a range of saturation frequencies and perform a voxel-by-voxel correction of the B_0_ shift.

Compared with the brain, B_0_ inhomogeneity is more severe in body CEST imaging which has larger field of view and heterogeneous tissue composition. To correct the B_0_ shift proper sequence and correction methods are required for reliable imaging performance. The B_0_ shift map could be acquired using the above Dixon method, fitted from the interpolated Z-spectra or a water saturation shift referencing (WASSR) [83] method. Dula et al. [48] implemented a simulation for the optimization of CEST detection using amide and GAG in fibroglandular breast tissues. Compared with other quantification metrics, MTRasym methods are usually more susceptible to B_0_ inhomogeneity.

Although not as critical as B_0_ correction for lower saturation power, B_1_ inhomogeneity affects signal quantification, especially when a large B_1_ is required for detecting fast-exchanging species. B_1_ corrections involves two steps: B_1_ mapping and the calculation of real contrasts. The B_1_ mapping methods include a double-angle method [84,85], Bloch-Siegert shift method [86], WASABI [87], and so on. The subsequent calculation can be mainly attributed to three different strategies. The first is to interpolate a value corresponding to rB_1_ (relative B_1_, defined as real B_1_/nominal B_1_) = 1 on the MTR-rB_1_ plane or Z-rB_1_ plane pixel by pixel [88]. The second is to fit the data on the MTR-rB_1_ or Z-rB_1_ plane with a selectively constructed function for each pixel group, divided based on the tissue types of T_1_ values [89,90]. The third is to perform Bloch-McConnell fitting on the Z-spectra and generate new Z-spectra with B_1_ values that are corrected from real ones to nominal ones [91].

### 3.3. Motion-Related Acquisition and Corrections

Human organs, such as the liver and lung, deform significantly with respiration, often causing severe motion artifacts in MRI without special designs. CEST usually applies a non-geometry specific 3D saturation pulse, thus the saturation is not affected much by motion but the water readout is. Therefore, a fast image readout was chosen, such as echo planar imaging (EPI), rapid imaging with refocused echoes (RARE), fast spin echo (FSE), and/or fast imaging with steady-state precession (FISP) [6,34,35,36]. As shown in Table 1, out of the three papers on liver and the two on lung, four used FSE [74,75,81,82] and one used EPI [73]. Besides a fast readout, respiration-gated design [81,82] or breath holding [73,74] are still required to reduce motion during acquisition.

Volumetric navigators (vNavs), a sequence block that is applied before the saturation pulses, can help to perform real-time motion correction for CEST [92]. As yet, vNavs have not been translated to body imaging, which may be studied in the future.

## 4. Applications

### 4.1. CEST Imaging of Breast Cancer

Breast cancer tops the list for cancer mortality for both women and the total population [93]. Our survey of the literature shows that approximately 2/7 of the clinical CEST/APT tumor studies involved breast cancer. This research has largely focused on two clinical aspects, the first involving the differentiation between the tumor subtypes and grades and secondly, for assessing the treatment responses.

#### 4.1.1. Differentiation of Malignant from Benign Lesions

**Figure 3:**
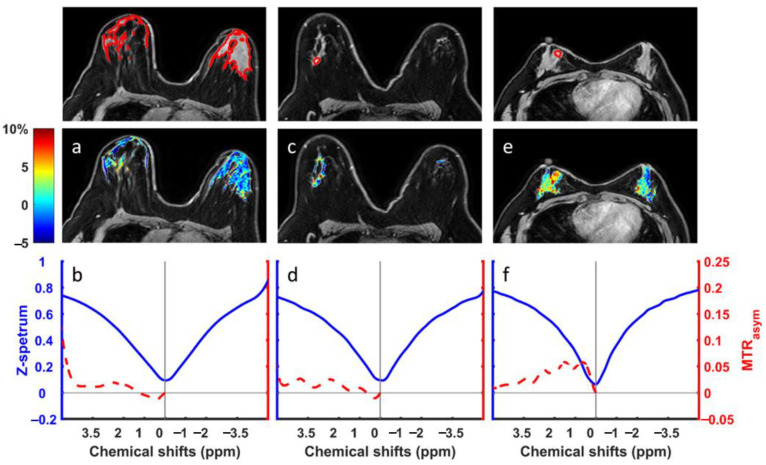
Hydroxyl CEST maps and ROI averaged Z-spectra (blue) and MTR_asym_ (red) for a healthy volunteer (**a**,**b**), invasive ductal carcinoma, not otherwise specified patient (**c**,**d**), and a triple-negative breast cancer patient (**e**,**f**). The CEST maps in (**a**,**c**), and (**e**) are overlaid on the reference water-only images. The panels above (**a**,**c**,**e**) show the corresponding ROIs in red, averaged across the fibroglandular tissues of both breasts (**b**); and averaged in the tumor areas as indicated by the ROIs (**d**,**f**). Reproduced with permission from John Wiley and Sons 2018 (DOI: 10.1002/mrm.27079) [45].

Schmitt et al. [51] reported that for three out of six patients, the regions of high CEST signal intensity matched well with tumor areas that were determined by DCE-MRI on a 3 T MRI. Significantly higher MTR_asym_ values at 1.8 ppm were detected in tumor tissue compared to normal breast tissue for these three patients. However, the high fat content that is associated with breast tissue may cause artifacts, resulting in a misdiagnosis. To remove fat interference, Zhang et al. [45] developed a CEST-Dixon sequence that was validated by breast cancer imaging at 3 T, which could well correct B_0_ inhomogeneity and obtain hydroxyl CEST maps at 1ppm (Figure 3). It was found that the MTR_asym_ at 1, 2, and 3.5 ppm for estrogen receptor (ER)-negative invasive ductal carcinoma (IDC) tissue was higher than those for ER-positive IDC, benign and normal tissues. However, there were no significant signal differences among the ER-positive IDC, benign, and normal tissues.

Loi et al. [40] employed relaxation compensated CEST signals for breast cancer characterization and quantified those using MTR_asym_. They found that amide CEST signals (3.5 ppm) as well as guanidyl CEST signals (2.2 ppm) were increased in tumor tissue compared to the normal appearing fibroglandular breast tissue of patients and healthy volunteers. Notably, APT and guanidyl CEST signals in fibroglandular tissue were not different between patients and healthy volunteers. However, Meng et al. [42] found that the MTR_asym_ (3.5 ppm) values of malignant tumors were significantly lower than those in benign lesions, also showing a weak correlation with pathological grade.

#### 4.1.2. Comparisons with Pathological Grades

Zaric et al. [44] compared MTR_asym_ values with the histological grades of breast tumors, reporting a significant increase in MTR_asym_ between Grade 1 and Grade 3 lesions. In contrast to other studies which assigned a constant offset (mainly 3.5 ppm), this paper did not use the same frequency offset for all patients. Instead, the highest values on the MTR_asym_ spectrum were selected, with the peak offset varying from 1.2 ppm to 3.55 ppm. In contrast, Meng et al. [42] found that the MTR_asym_ (3.5 ppm) values did not provide good correlations with the pathological grade (r = 0.371). Zaric et al. [44] further studied the relationship between MTR_asym_ values and cell proliferation, and found a strong positive correlation between MTR_asym_ and the Ki-67 proliferation index. However, Loi et al. [40] reported that Ki-67 only moderately correlated with the amide and the guanidyl CEST signals. Zhang et al. [45] used CEST-Dixon to characterize different types of breast cancer tissue and found that in the three frequency ranges that were studied, 1 ppm CEST signals (MTR_asym_) were the highest in ER-negative IDC cases, exhibiting the highest correlation with Ki-67 and the largest differences among each of the tissue groups.

#### 4.1.3. Assessment of Treatment Responses

To assess the response to neoadjuvant chemotherapy (NAC), Klomp et al. [50] imaged breast tissues with 3 T APT-MRI, and showed that the APT values that were calculated by the Lorentzian difference increased during disease progression while conversely decreased in patients that showed partial or complete responses. Krikken et al. [46] further tested the ability of CEST to evaluate early response to NAC in breast cancer patients. For six out of the ten lesions that were analyzed, significant differences were found between the APT signals that were calculated by three-pool Lorentzian fitting acquired pre- and post-NAC. However, one of the two pathologically validated complete response cases showed no significant difference in pre- and post-NAC APT signals. Moreover, different pathological responses to NAC treatment showed no significant differences in changes in APT signals. Zhang et al. [41] found that quantitative APT_W_ MRI depended on optimizing acquisition saturation powers and analysis methods, and also monitored the treatment effects but did not differentiate participants with triple-negative breast cancer who had a pathologic complete response (pCR) from those with non-pCR.

One of the consequences of breast cancer resection involves lymphedema in the patients’ upper extremities, which may be relieved by lymphatic mobilization therapy. Donahue et al. [47] found APT signals (both standard asymmetry and Lorentzian asymmetry) had no significant difference between the right and left arms of healthy controls but values increased in the patient’s arm that was affected by lymphedema. Crescenzi et al. [43] found that the proton transfer ratio (PTR, defined as 1–Z’, where Z’ is the Z-spectra after B_1_ correction) APT significantly correlated with T_1_ and BMI (body-mass-index) in controls, and the lymphedema stage in breast cancer treatment-related lymphedema (BCRL) participants. The post-therapy PTR of APT significantly increased in the affected arm of BCRL participants, consistent with the treatment effects that were from mobilized lymphatic fluid.

### 4.2. Pelvic Tumors

Pelvic tumors include those of the cervix uteri, corpus uteri, ovary, and prostate, and all ranked among the top ten in the list of cancer incidences and mortality rates worldwide in 2020 [93]. All of the studies were performed using 3 T MR scanners.

#### 4.2.1. Cervical Cancer

**Figure 4:**
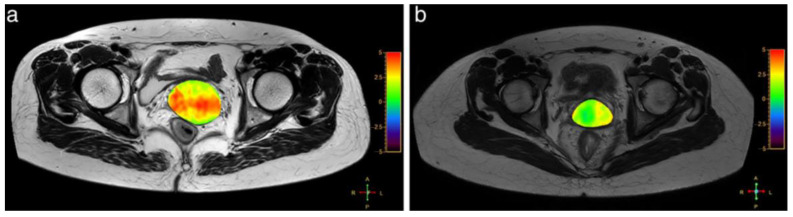
(**a**) APTw image of a 47-year-old woman with cervical squamous cell carcinoma; the APT value was 2.68. (**b**) APTw image of a 46-year-old woman with a normal cervix; the APT value was 1.76. Reproduced with permission from John Wiley and Sons 2019 (DOI: 10.1002/jmri.26710) [54].

All cervical cancer studies reported used MTR_asym_ (3.5 ppm), i.e. APTw image, for CEST quantification. He et al. [54] compared 75 patients with cervical lesions (mostly squamous cell carcinoma and adenocarcinoma) against 49 healthy volunteers, finding that the APT values of cervical cancer and normal cervical stroma showed highly significant differences (*p* < 0.0001). Typical images were shown in Figure 4.

In a study of cervical squamous carcinomas (CSCs), Sun et al. [53,54] found that the APT values (MTR_asym_(3.5ppm))of the squamous cell carcinoma of the cervix (SCCC) were higher than normal cervical stroma. Significant differences were similarly found in the APT values between the moderately- to well-differentiated CSC and poorly differentiated CSC cases. The APT values for histologic Grades 1 to 3 were also significantly different.

Using a scanner from a different manufacturer than above studies, Meng et al. [52] found that APT values in cervical cancer cases were higher than those of normal cervixes. The APT value of the cervical adenocarcinoma group was higher than that of the CSC group. The APT values were found to gradually increase between the high-, middle-, and low-differentiation cases of CSC, but only statistically significant differences were measured between the high- and low-differentiation groups (*p* < 0.05). No significant difference was found between the Grade 1 and Grade 2 tumors, or between well- and moderately-differentiated cancers in all of the above studies.

#### 4.2.2. Endometrial Carcinoma

A total of five groups have contributed reports that were involving cervical cancer and endometrial carcinomas along with healthy uterine tissues. APT values changed with cancer type [56], histology grade [60], proliferation, as well as menstrual cycle. The APT method was co-studied with other fMRI methods [57].

**Figure 5:**
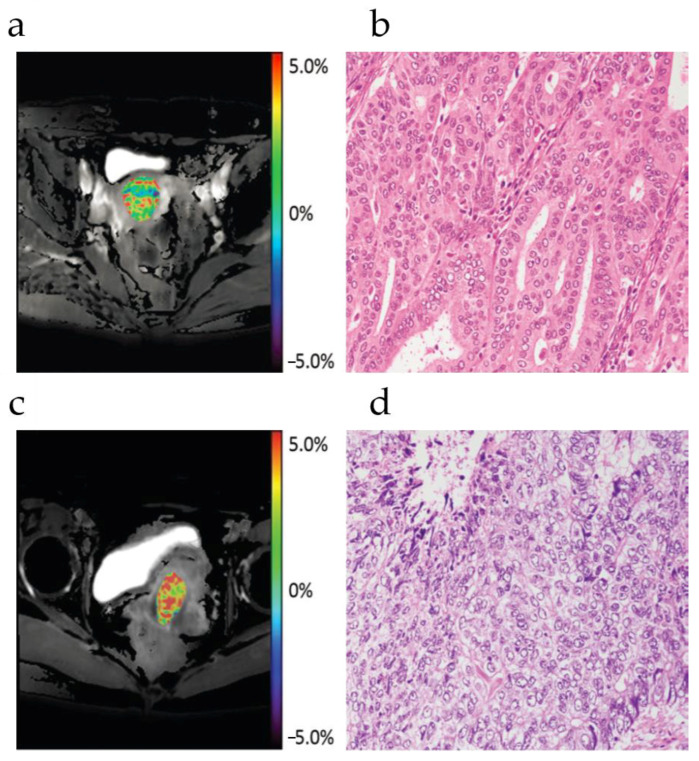
Images of a 71 year-old woman (patient 1) with Grade 1 EEA and a 50-year-old woman (patient 2) with Grade 3 EEA. APT image of EEA fused with fat-suppressed proton density–weighted-imaging of patient 1 (**a**) and patient 2 (**c**), as well as the microscopic image of hematoxylin-eosin(H&E) staining of EEA (original magnification, ×200) of patient 1 (**b**) and patient 2 (**d**). The tumors of the two patients show inhomogeneous SIs on the APT image, and the averaged APT SIs obtained by the two readers were 1.7% for patient 1 and 3.7% for patient 2, respectively. The microscopic image of patient 1 shows the proliferation of well-differentiated EEA cells, arranged in irregular glands and tubules; The cell morphology, density and distribution features indicate Grade 1 EEA. The microscopic image of patient 2 shows the proliferation of moderately- to poorly-differentiated EEA cells, arranged in solid and glandular patterns; The cell morphology, density and distribution features indicate Grade 3 EEA. Reproduced with permission from Radiological Society of North America 2018 (DOI: 10.1148/radiol.2017170349) [60].

Ochiai et al. [56] evaluated the efficacy of APT imaging in the differentiation of type I and type II uterine endometrial carcinoma in a 33-patient study. Results show that APT imaging has the potential to determine the type of endometrial cancer. Takayama et al. [60] compared APT values with the histological grades of endometrioid endometrial adenocarcinoma (EEA), the most common type of endometrial carcinoma. With a Spearman correlation coefficient of 0.55 reported, the average APT values of Grade 1 to 3 EEA were 2.2% ± 0.2, 3.2% ± 0.3, and 3.7% ±0.3, respectively. With the typical APTw images and the corresponding histology pictures shown in Figure 5, population-wise the APT values of Grade 3 EEA cases were significantly higher than those of Grade 1 (*p* = 0.01), but other pairwise comparisons did not reveal any significant differences (*p* = 0.06–0.51). He et al. [58] compared the APT values of low-proliferation (Ki-67 < 30%, *n* = 8) and high-proliferation cases (Ki-67 > 30%, *n* = 14) of type I endometrial carcinoma, showing there was a moderate positive correlation between the Ki-67 labeling index and APT values (r = 0.583, *p* = 0.004). Sun et al. [59] investigated 20 healthy women of childbearing age and found that the APT values did not differ significantly between the endometrium and myometrium during any phase. In each uterine structure, the APT values decreased from the secretory phase to the proliferative phase and reached the lowest values in the menstrual phase. However, the APT values did not differ significantly between the menstrual phase and proliferative phases. Inter-individual variation in the APT values for a given zone or phase ranged from 1.86% to 2.75%. This study indicated that changes that are caused by the menstrual cycle should be considered for CEST imaging of the uterus.

Meng et al. also studied endometrial carcinoma (EC) by using APT and the other methods [57]. The authors found that the APT values were significantly less in low-risk cases compared to those of higher risk, and moreover, that the APT values, diffusion coefficient (D), and mean kurtosis (MK) were independent predictors of risk stratification. Nevertheless, the combination of these three parameters was able to better identify low- and high-risk groups compared to the individual measures.

#### 4.2.3. Prostate Cancer

CEST has been used in prostate cancer classification and tumor characterization. Jia et al. [65] found that MTR_asym_ values in prostate cancer ROIs were significantly higher than for those in the peripheral zone benign regions. This is an encouraging indication that APT MR imaging may be feasible for prostate cancer detection and has the potential to distinguish cancerous from non-cancerous tissue. Takayama et al. [64] studied the relationship between APT values and prostate cancer Gleason scores (GS), finding that the mean ± SD APT values varied for GS-6 (2.48 ± 0.59), GS-7 (5.17 ± 0.66), GS-8 (2.56 ± 0.85), and GS-9 (1.96 ± 0.75), respectively. This is a progressive grading score, but the APT value of the GS-7 group was highest, with significant differences measured between the GS-6 and GS-7 groups and the GS-7 and GS-9 groups (*p* < 0.05). Yin et al. [61] also found that diffusion kurtosis imaging (DKI) and APT imaging are valuable in the diagnosis of prostate cancer (PCa) and demonstrated a strong correlation with the Gleason Score, which had great significance in the risk assessment of PCa.

#### 4.2.4. Ovarian Cancer

AcidoCEST can be used to measure the extracellular pH (pHe) of human tumors, which may reflect the metabolic status of the tumors, or be used to detect tumors among normal tissue backgrounds. Jones et al. [66] found that in one patient with metastatic ovarian cancer, the average pHe value of three adjacent tumors was 6.58, whereas the average pHe of the kidney was 6.73. Bloch equations were used to fit the CEST spectra to get a pHe value of the imaging tissue. An FDA-approved clinical contrast agent for CT clinical studies named Iopamidol (Isovue, Bracco Imaging, Inc., Milan, Italy) was used in this study for CEST imaging, whose signal was linearly correlated with pH values. Lorentzian fitting was also used in this study.

### 4.3. Digestive Tumors

Digestive tumors include rectal, liver, and salivary cancers. Both rectal and liver cancers appear in the top 10 list of cancer incidence and mortality rates worldwide in 2020 [93].

#### 4.3.1. Rectal Cancer

**Classification and tumor grading:** Chen et al. [67] found that the combination of APTw and DWI may serve as noninvasive biomarkers for evaluating and identifying responses to neoadjuvant chemoradiotherapy in locally advanced rectal cancer patients.

Nishie et al. [71] studied 22 rectal cancer patients and reported significant differences in the average MTR_asym_ of tumors with diameters more than 5 cm and less than 5 cm. There were also significant differences between MTR_asym_ in moderately- versus well-differentiated adenocarcinomas. In contrast, the apparent diffusion coefficient (ADC) could not distinguish between groups that were classified by any pathological factor. Xian Liu’s group [68] applied APT MRI and DWI into the assessment of two important prognostic factors of rectal adenocarcinoma, which were the p53 status and Ki-67 index. The histological grade, T stage, and N stage were also evaluated. It was found in 43 patients that high-grade tumors, tumors of more advanced stage, and tumors with lymph node involvement showed significantly higher mean MTR_asym_ values. In contrast, ADC values were also significantly different in terms of different pT stages, but not in terms of pN stages or histological grades. Regarding the prognostic markers, p53 positive status was correlated with higher mean MTR_asym_ values, but not with ADC values. Notably, both MTR_asym_ and ADC values were significantly different between tumors with low and high Ki-67 expression status. Liu et al. [69] also conducted a study to compare the utility of APT MRI and that of diffusion kurtosis imaging (DKI) in predicting several pathologic factors for rectal adenocarcinoma, which were WHO grade, pT stage, pN stage, and EMVI status. It was found that significantly higher mean APTw signal intensity (SI) was correlated with high-grade tumors, as well as T3 stage tumors with lymph node metastasis or EMVI-positive status. Compared with kurtosis, diffusivity, and ADC, APTw SI was a better discriminating index of tumor grading.

Various studies compared the CEST parameters with the Ki-67 proliferation index in histology. The CEST-Dixon sequence was used at 3 T for rectal tumors [68]. The mean APTw had a positive correlation with Ki-67. It also had a significantly higher diagnostic ability for the differentiation of the high Ki-67 expression tissue group than ADCmean. However, this conclusion may not apply to mucinous adenocarcinoma or heterogeneous tumors [71]. Another study on 7 T calculated CEST MTR_asym_ values at three different offsets for breast cancer also reported a strong positive correlation between the largest MTR_asym_ value in each patient and the Ki-67 index. However, this study did not use the Dixon method for fat suppression. Instead, a frequency-dependent water excitation pulse was used [44].

**Assessment of treatment response:** Nishie et al. [70] examined 17 patients with locally advanced rectal cancer who underwent neoadjuvant chemotherapy. According to the treatment responsiveness, the mean MTR_asym_ of lesions of patients showing limited responses was significantly higher than those who responded to therapy. Analysis of the predictive ability of MTR_asym_ for tumor responses showed values of 75% sensitivity and 100% specificity.

#### 4.3.2. Liver

**Figure 6:**
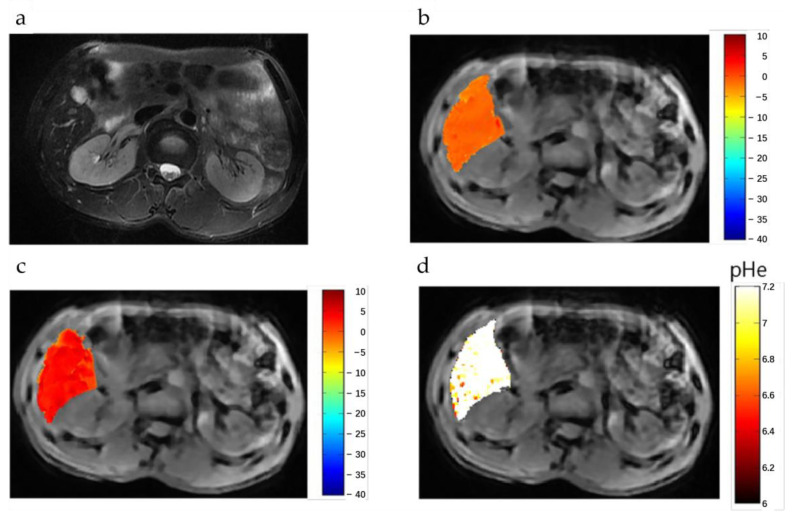
pHe values of hepatic hemangioma using dual-power CEST MRI. (**a**) A representative image of a patient with hepatic hemangioma.After injection of a CT agent (Ioverol), CEST images acquired with flip angles of 60° (**b**) and of 350° (**c**), respectively. (**d**) The pHe map for hepatic hemangioma; the CEST pHe was consistent with the surrounding liver tissue, confirming the hemangioma to be benign. Reproduced with permission from Frontiers 2020 (DOI: 10.3389/fonc.2020.578985) [72].

**Tumor detection**: Tang et al. [72] performed ioversol-based pH mapping on a 3 T scanner. The CEST-related signal was separated using MTR_asym_ at 4.3 ppm and the pH effect was measured by a specially designed ratiometric value that was calculated from the signals that were obtained at two flip angles (Figure 6). The logarithm of the ratiometric value was found to be proportional to pH values in vitro and thus the pH values can be estimated. In a 15-patient study, the estimated pH values were significantly lower in hepatic carcinomas (6.66 ± 0.19) than in the normal liver tissue (7.31 ± 0.12; *p* < 0.0001). However, in a 5-patient study, no significant differences (*p* = 0.5587) were found between hepatic hemangioma (7.34 ± 0.09) and normal tissues (7.37 ± 0.08).

**Predicting histological grade:** Lin et al. [74] found that the APTw imaging is a useful imaging biomarker that complements DWI for the more accurate and comprehensive hepatocellular carcinoma (HCC) characterization. Both APTw and DWI had good diagnostic performance in differentiating the high- from the low-grade HCCs, with areas under the curves of 0.814 and 0.745, respectively. Moderate correlations existed between the APTw values and histological grades, as well as the ADC values and histological grades.

**Assessment of treatment response:** Jia et al. [73] constructed a protocol to predict the intermediate-stage hepatocellular carcinoma response to trans-arterial chemoembolization, in which APT imaging at 3 T was an important step. APT was quantified with MTR_asym_. In the three groups (i.e., tumor, peritumoral, and normal tissues), the APT signals were in good agreement within each group and significantly different between groups.

#### 4.3.3. Salivary Gland Tumors

**Figure 7:**
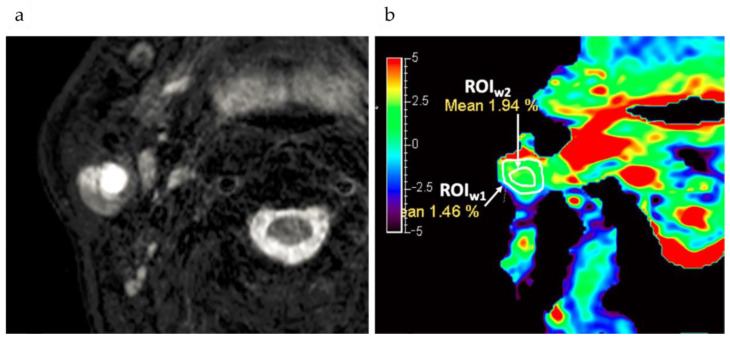
An APTw example showing excellent image quality. A round lesion was found in the right parotid gland of a 65 year-old male, as shown on T2WI (**a**). The corresponding APTw image of the same slice is shown in (**b**). The image quality for this APTw map in terms of integrity and hyperintensity artifacts on the lesion was scored as 4 and 4, respectively. The lesion was considered as being in the trustable group. The average APTw value of the lesion was 1.94% for the ROIw2, drawn avoiding the surrounding hyper intensity artifacts in the ROIw1. The arrows mean APTw values in ROI. Reproduced with permission from AME 2021 (DOI: 10.21037/qims-20-675) [75].

**Tumor detection:** Yu et al. [78] found that APTw MRI is feasible for use in head and neck tumors and is a valuable imaging biomarker for distinguishing malignant from benign lesions. Yuan et al. [79] also found that APTw MRI was feasible for use in the head and neck regions at 3 T in clinical applications. Chen et al. [75] found that most APTw images of tumor lesions in parotid glands had an acceptable image quality (Figure 7), hence were feasible for diagnostic use.

**Comparison with other methods** Takumi et al. [76] reported that MTR_asym_ values of APT in malignant lesions were significantly higher than for benign lesions (*p* = 0.047). However, no significant differences were found in ADC or tumor blood flow (TBF) between benign and malignant lesions in the same patients. The accuracy of the three parameters combined was significantly higher than that of each parameter alone. It was concluded from this study that the combination of ADC, TBF, and MTR_asym_ was more helpful in differentiating malignant from benign salivary gland lesions. Bae et al. [77] found in a 38 subjects study that the APTw signals for major salivary gland tumors were significantly higher in malignant tumors than in benign ones in terms of maximum, mean, and median measures. Notably, the diagnostic performance of APTw signals was superior compared with the combination of DWI and DCE-MRI, indicating that APTw-MRI could benefit the differential diagnosis of major salivary gland tumors in the clinic.

### 4.4. Lung Cancer

Lung cancer is on the top 10 list of the cancer incidence and mortality rates worldwide in 2020 [93].

**Differentiation of malignant from benign pulmonary nodules:** A study of 82 patients with pulmonary nodules by Ohno et al. [81] measured APTR using MTR_asym_ at 3.5 ppm. It was shown that although the sensitivity of ADC was significantly higher than that of APTR (*p* = 0.002) along with FDG-based SUV_max_ measurements (maximum value of standard uptake value; *p* = 0.004), the specificity of APTR and SUV_max_ was significantly higher than for ADC (*p* < 0.05). Moreover, the sensitivity of combined APTR with SUV_max_ was significantly higher than either APTR (*p* = 0.001) or SUV_max_ (*p* = 0.002) alone. Additionally, the specificity and accuracy of combined APTR and SUV_max_ were significantly higher than for ADC (specificity: *p* = 0.002, accuracy: *p* = 0.008). Together, these data confirm the effectiveness of CEST in differentiating benign from malignant nodules.

**Assessment of treatment response:** Jones et al. [80] developed a retrospective respiration-gated method that was based on phantom studies as well as three patients with lung cancer. The method was then applied to four lung cancer (or mesothelioma) patients to assess their reactions after radiation therapy and/or chemotherapy. The results indicated more precise measurements using retrospective respiration-gated analysis in all cases compared to non-gated analysis methods, showing this approach can improve the CEST MRI evaluations of tumors and organs that are affected by respiratory motion. The preliminary clinical study showed a large increase in MTR_asym_ at 3.5 ppm after radiation therapy, a small increase or decrease in MTR_asym_ after chemotherapy, and mixed results with combined chemoradiation therapy, suggesting that the CEST MRI may be more sensitive to radiation therapy than to chemotherapy.

**Figure 8:**
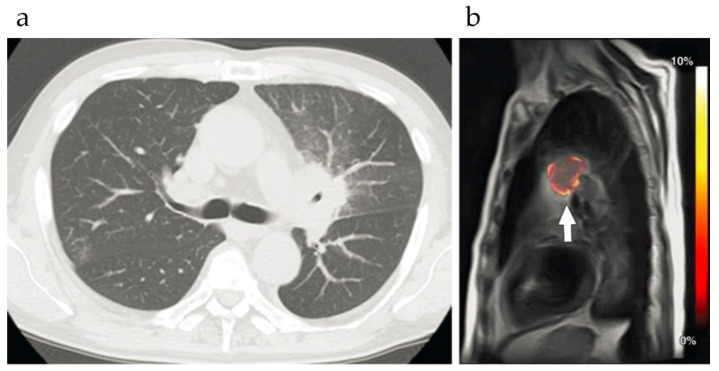
Squamous cell carcinoma in a 66 year-old man. (**a**) Axial thin-section CT image (lung window setting) shows a left hilar mass, with obstructive pneumonia. (**b**) Sagittal APT-weighted CEST MR map shows a left hilar mass (arrow) with low MTR_asym_ (at 3.5 ppm) and relatively high MTR_asym_ (at 3.5 ppm) of the surrounding obstructive pneumonia. Reproduced with permission from Radiological Society of North America 2016 (DOI: 10.1148/radiol.2015151161) [82].

**Characterization of thoracic lesions:** Ohno et al. [82] studied malignant and benign thoracic lesions in 21 patients and found that MTR_asym_ for malignant tumors was 3.56% ± 3.01, significantly higher than that for benign lesions (0.33% ± 0.38, *p* = 0.008). Lung cancer MTR_asym_ values were significantly lower than for other thoracic malignancies (2.16% ± 1.41 versus 6.71% ± 3.46, respectively; *p* = 0.005). Moreover, among lung cancers, MTR_asym_ for adenocarcinomas was significantly higher, than for squamous cell carcinomas shown in Figure 8 (2.88% ± 1.13 versus 0.71% ± 0.17, respectively; *p* = 0.02).

### 4.5. Comparison with Other Functional MRI Methods

In many above studies, the diagnosis capability of APTw-MRI was compared with other functional MRI methods including DWI and contrast-enhanced images. To further clarify, Table 2 summarized the basic principle, quantification parameters, application in tumor imaging, as well as the advantages and disadvantages, for the three MRI-based imaging methods.

## 5. Discussion and Future Prospects

### 5.1. Advantages of CEST in Cancer Detection

CEST is a newly developed clinical MR imaging method. The key advantages of CEST imaging include:

(1) As a sensitive chemical-shift based method, the spatial resolution could be close to the standard MR images.
(2) Contrast could be turned “on” and “off” by the acquisition sequence, and “multi-color” imaging could be achieved in parallel with optical imaging.
(3) CEST can detect both endogenous and exogenous agents. When this method detects the endogenous contents of lipids, mobile proteins/peptides, glycans, as well as small metabolites in tissue itself, CEST does not need to consider the delivery and targeted efficiency of agents. In addition, the surrounding normal tissue could be employed as an internal reference.
(4) Body imaging is easier for using CEST agents due to the lack of blood-brain barrier.

### 5.2. Challenges for Implementing CEST in the Clinic

However, there still are some challenges to be met for the future development and implementation of CEST.

(1) Saturation power and imaging time

For more practical clinical usage, CEST needs to be implemented with less saturation power and reduced imaging times. To meet the FDA-guided specific absorption rate requirements [40], CEST applications in humans may have a limited saturation pulse duration or duty cycle or RF amplifier for low power deposition. New excitation sequences could, therefore, potentially resolve the tradeoff between imaging quality and power usage.

With regard to shortening the scan times, there are at least two possible pathways: to reduce the number of scans that are necessary, or to acquire more scans in a defined time window. A short scan time strategy called SAFARI (a sequence of saturation with frequency alternating RF irradiation) has been reported as requiring only three image acquisitions while maintaining the specificity of CEST detection [95]. The MTRdouble method as proposed by Gochberg’s group [96] requires as few as three data points, which is more rapid than methods requiring a complete Z-spectrum. The multi-echo length and offset varied saturation (MeLOVARS) technique uses the idea of Look-Locker and obtains several echoes in each repetition period [97]. More saturation techniques such as these are needed to aid the development of CEST usage in the clinic.

(2) B_0_/B_1_ fluctuation effects

The CEST signal is sensitive to $B_{0}/B_{1}$ fluctuations or the movement artifact of organs, such as bowel motions [14]. Besides saturation techniques, data analysis methods also need to focus on removing the background effects that are caused by MT or NOE. Furthermore, optimized and standardized scan protocols for CEST MRI are necessary for clinical usage [33].

One point that needs to be raised is that the uniformity of the main magnetic field, $B_{0}$, is very important for CEST imaging, especially in vivo imaging. In addition, the uniformity of the saturation field strength, $B_{1}$, also affects CEST quantification especially for fast-exchanging protons, since CEST saturation efficiency is determined partially by $B_{1}$. On one hand, higher magnetic fields could achieve better SNR and frequency resolution, which is beneficial for CEST imaging. On the other hand, $B_{0}$ and $B_{1}$ inhomogeneity will increase under higher field strengths, therefore, proper $B_{0}$ and $B_{1}$ correction needs to also be considered. Notably, the larger field of view that is associated with body imaging is more challenging than brain imaging due to both the heterogeneous tissue composition and the $B_{0}$ and $B_{1}$ field inhomogeneity.

(3) Artifact elimination

The potential sources of artifact that are affecting the quantitative and qualitative discrimination of tissues are numerous and include fat or air in the imaging ROI; tissue movement during imaging caused by breathing, bladder filling, bowel movements; as well as errors caused by menstruation. As previously discussed, CEST-Dixon has been used to suppress fat artifacts [45]. Some motion-correction methods have also been developed [98,99,100,101], but there is still room for improvement in the scope of application and the correction performance. Thus, further studies are needed to improve artifact elimination.

(4) Interpretation of the results

For clinical usage, the relationship between CEST values and traditional histology characteristics need to be studied more thoroughly. Several studies [52,60] have reported that MTR_asym_ values were related to the histological grade or could differentiate the grades of tumors. However, only MTR_asym_ values of Grade 3 (or moderate- to well-differentiated) lesions were significantly different from Grade 1 or 2 (poorly-differentiated) lesions. The difference between MTR_asym_ values of Grade 1 and 2 (or moderately- and well-differentiated) lesions or Grades 2 and 3 were not significant.

### 5.3. Future Prospects

As in Figure 1, CEST imaging of non-brain tumors is a fast-growing field. Except for endogenous APTw imaging, advanced sequences and the quantification of multiple CEST and NOE sources are under development [101]. There are also two types of clinically approved agents, glucose and CT agents, that have been investigated under high-field pre-clinical scanners (Table 3).

For non-APTw imaging, Zijl et al. [35] quantified the hydroxyl proton signal at 1 ppm using MTR_asym_ to detect the relative content of glycogen in mouse livers. Zhou et al. [34] found that the intensity of the NOE signal at −1 ppm was also contributed by glycogen (thus namely glycoNOE) and validated this notion in mouse livers after fasting and glucagon injection. Together these studies highlight the potential of liver tumor detection by imaging glycogen using CEST.

GlucoCEST MR was used in a prostate study by Kim et al. [63], who found that the Gaussian hyperglycaemic clamp infusion that is based on the DeFronzo method demonstrated higher efficiency and stability of glucose delivery as compared to manual determination of glucose infusion rates. Dynamic glucose enhancement (DGE) signal sensitivity was found to be dependent on T_2_, $B_{1}$ saturation power, and integration range. Motion correction and $B_{0}$ field inhomogeneity correction are crucial to avoid mistaking signal changes for a glucose response while field drift is a substantial contributor. However, after $B_{0}$ field drift correction, no significant glucoCEST signal enhancement was observed in tumor regions of all patients. Thus, glucoCEST at 3 T is not yet practicable in body regions and physical movements and the effects of $B_{1}$ and $B_{0}$ made the originally small glucoCEST signal difficult to detect. Nasopharyngeal [102] and pancreatic cancers [106,107,108,109,110] were studied in animal models. CEST was used to detect tumor [108,109,110] and characterize the tumor tissue dynamically during therapy [102,106,107]. The magnetic strength of the scanner that was used in the studies of the pancreas was still too high for clinical usage.

Nevertheless, CEST imaging of non-brain tumors, either with or without a contrast agent, is a promising clinical tool that is useful for tumor diagnosis and prognosis.

## Figures and Tables

**Table 1:** CEST acquisition and analysis methods/parameters in non-brain tumor imaging *.

CT	Year	SN	Saturation Preparation			Resolution (mm^3^)	AT (s)	Readout Sequence	Quantification Metrics	Study
			Pulse Type	T_sat_ (ms)	B_1_ (μT)					
Breast	2021	51	PT	3500 2000	0.9 2.0	1.2 × 1.2 × 5	258	TSE	LF MTR_asym_	[41]
	2020	121	PT	2000	2.0	2.5 × 2.5 × 4	260	EPI	APTw	[42]
	2020	17	PT	5600	0.6, 0.9	0.7 × 0.6 × 4.2	1200	2D-GRE	LF; AREX	[40] ^#^
	2020	29	PT	75	2	1.8 × 1.47 × 5.5	360	EPI	PTR’_APT_, PTR’_NOE_, MTR’_asym_, AREX’	[43]
	2019	21	PT	500	1	1.7 × 1.7 × 4	810	3D-GRE	MTR_asym_	[44] ^#^
	2018	15	CW	500	1.2	2 × 2 × 5	70, 146	2D-Dixon	MTR_asym_	[45]
	2018	9	PT	4 s	2	2.3 × 3.0 × 6.8	295	GRE	LF	[46] ^#^
	2016	15	PT	75	1	1.0 × 1.3 × 3.0	N/A	3D-GRE; Dixon	LF	[47]
	2015	10	PT	25	1	1.0 × 1.0 × 6.0	N/A	3D-GRE	LF	[48] ^#^
	2013	6	PT	4000	3	3 × 3 × 6	300	Turbo field echo	LF	[49] ^#^
	2013	13	PT	962.5	0.5	2.5 × 2.5 × 5.0	402	N/A	LF; Z-spectra	[50]
	2011	6	PT	100	1.5	2.7 × 1.5 × 3.0 6.9 × 1.5 × 3.0 1.4 × 1.5 × 3.0	N/A	3D-GRE; SPAIR	MTR_asym_;	[51]
Cervix	2019	76	PT	2000	2.0	0.3 × 0.3 × 5.0	156	EPI	APTw	[52]
	2019	32	PT	2000	2.0	2.0 × 2.0 × 5.0	406	SPIR; 3D-TSE	APTw	[53]
	2019	124	PT	2000	2.0	2.5 × 2.5 × 2.5	453	SPIR; 3D-TSE	APTw	[54]
	2019	31	PT	2000	2.0	2.0 × 2.0 × 5.0	406	SPIR; 3D-TSE	APTw	[55]
Uterus	2021	33	PT	2500	1.7	2.3 × 1.9 × 5.0	246	TSE	MTR_asym_	[56]
	2021	80	PT	500	2.0	2.8 × 2.8 × 5.0	156	2D-EPI	APTw	[57]
	2021	54	PT	2000	2.0	2.5 × 2.5 × 5.0	453	3D-TSE; SPIR	APTw	[58]
	2019	20	PT	2000	2.0	2.0 × 2.0 × 5.0	406	3D-TSE Dixon; SPAIR	APTw	[59]
	2018	32	PT	500	2.0	1.8 × 1.8 × 5.0	140	2D-GRE	APTw	[60]
Prostate	2021	100	PT	500	2.0	2.2 × 2.2 × 5.0	156	EPI	APTw	[61]
	2019	7	PT	4800	0.92	2.2 × 2.2 × 4	342	TSE; SPIR	LF; MTR_asym_	[62]
	2019	1	PT	40	2.5	2.18 × 2.22 × 10.00	170	TSE; SPIR	Z-spectra; glucoCEST signal	[63]
	2016	141	PT	500	2.0	1.8 × 1.8 × 5.0	140	2D-GRE	APTw	[64]
	2011	12	PT	496	3.8	1.8 × 2.2 × 6.0	214	TSE	APT ratio	[65]
Ovary	2017	1	PT	991 2000	1.5	N/A	1425	Turbo-FLASH	AcidoCEST; LF	[66]
Rectum	2021	53	CW	2000	2	1.8 × 1.8 × 5	N/A	3D-TSE Dixon	MTRasym	[67]
	2020	43	qCW	2000	N/A	1.8 × 1.8 × 5.0	270	3D-TSE Dixon; SPIR	APTw	[68]
	2020	61	CW	2000	2.0	1.8 × 1.8 × 5.0	270	TSE; Dixon	APTw	[69]
	2019	17	PT	500	2.0	1.8 × 1.8 × 5.0	140	TSE	MTR_asym_	[70]
	2018	22	PT	500	2.0	1.8 × 1.8 × 5.0	140	TSE	MTR_asym_	[71]
Liver	2020	20	PT	28	0.2, 1.15	1.88 × 1.88 × 5.0	391	GRE	MTR_asym_	[72]
	2020	56	N/A	N/A	N/A	3.1 × 3.1 × 5.0	N/A	EPI	MTR_asym_	[73]
	2019	32	PT	830	2	1.0 × 1.5 × 6	261	2D-TSE	MTRasym	[74]
Salivary gland	2021	36	PT	2000	2.0	2.5 × 2.5 × 5.0	160	3D-TSE	MTR_asym_	[75]
	2021	42	PT	2000	2.0	1.8 × 1.8 × 5.0	112	2D-GRE	APTw	[76]
	2019	38	PT	70	2.0	2.0 × 2.5 × 6.0	245	3D-EPI	APTw	[77]
Head & neck	2019	29	PT	830	2	2.2 × 2.2 × 6	261	TSE	MTR_asym_	[78]
	2014	10	CW	200	2.0	2.0 × 2.0 × 4.0	120	TSE	APTw	[79]
Lung & Thoracic	2017	7	CW	200	1.0	4.7 × 4.7 × 20.0	180	Steady-state precession	MTRasym	[80]
	2017	82	PT	400	1.0–2.0	1.2 × 1.4 × 15.0	600	2D-half Fourier TSE	APTw	[81]
	2016	21	PT	400	1.0–2.0	1.2 × 1.4 × 15.0	600	Half-Fourier TSE	APTw	[82]

* Only CEST related properties of the studies are listed. ^#^ The magnetic field intensity of these studies was 7 Tesla, while that of the other studies was 3 Tesla. CT = cancer type; SN = subject number; Tsat = saturation time; AT = acquisition time; PT = pulse train; TSE = turbo spin echo; LF = LF; MTR_asym_ = magnetization transfer ratio asymmetry; EPI = echo planar imaging; APTw = APT-weighted; AREX = apparent-exchange-dependent-relaxation; CW = continue wave; GRE = gradient echo; SPAIR = spectral attenuated inversion recovery; PTR = proton-transfer-ratio;’ = metrics corrected for B_1_ efficiency; SPIR = spectral pre-saturation with inversion recovery; TurboFlash = Turbo Fast Low-angle Shot; qCW = quasi-continuous wave.

**Table 2:** Comparison of MRI-based imaging methods.

Imaging Type	APTw-MRI	DWI-MRI	DCE-MRI
Full name	Amide proton transfer-weighted MRI	Diffusion-weighted imaging MRI	Dynamic contrast-enhanced MRI
Target	amide proton constituents	Cell density, tumor microstructure	Contrast enhancement kinetics
Imaging principle	Based on the effect of CEST between free water and mobile proteins or peptides backbones; amide proton constituents abundant in tumors.	Measuring the random Brownian motion of water molecules within a voxel of tissue. Highly cellular tissues exhibit lower diffusion coefficients.	Uses the T_1_ relaxation characteristics of gadolinium contrast agents to model the pharmacokinetic distribution of contrast between the vasculature and interstitial space
Parameter	APT signal intensity (APT SI)	Apparent diffusion coefficient (ADC)	Time-intensity curve (TIC); k_ep_ (the exchange of the contrast agent between the two compartments)
Clinical application in tumor imaging	Diagnosis tumor, predict tumor response to treatment, assessment of prognostic factors	Tumor grading, diagnosis and prognosis; Assessing the proliferation status of several cancers	Assess the therapeutic response of tumor. Important for the clinical evaluation of EEA, especially for assessment of the depth of myometrial invasion. [60]
Advantages	Needs no exogenous contrast agent; Quantitative imaging parameters correlate with histopathology or oncogenic protein markers, such as p53 and Ki-67 index [94]	Effective in the differentiation with high diagnostic accuracy	The golden standard of neovascularization; Effective in the differentiation with high diagnostic accuracy;
Disadvantages	APT imaging is often prone to artifacts resulting from system Instability [42]	ADC diagnostic and prognostic capacity is reduced by the complicate components in tumor interstitial regions	Needs exogenous contrast agent; Contrast enhancement kinetics in tissue depend on several factors such as microvessel density and vascular permeability, which are not pathognomic for some tumors like breast tumors [51]

**Table 3:** Preclinical studies.

Body Part	Year	MS (T)	Saturation Pulse		Resolution (mm^3^)	AT (s)	Technical Novelty	Study
			T_sat_ (s)	B_1_ (μT)				
Nasopharyngeal	2021	3.0	0.8	2	1.25 × 1.25 × 7	381	MTR_asym_	[102]
Breast	2021	7	5	1.5	0.3125 × 0.3125 × 1.5	128	Contrast agents: voluven and dextran 70	[103]
	2019	7	5	1.5	0.39 × 0.39 × 4	793	Integrating CEST contrast agents into nanocarriers	[104]
	2017	7	5	1.5	0.234 × 0.234 × 1.5	~605	Pharmaceutical excipients as contrast agents	[105]
Pancreas	2020	14	1	3	0.2 × 0.2 × 1	1800	Rare sequence; WASSR;	[106]
	2019	14	1	3	0.2 × 0.2 × 1	1140	Rare sequence; WASSR;	[107]
	2019	11.7	3	1.8	0.4 × 0.4 × 1	300	Contrast agent; RARE sequence; WASSR;	[108]
	2019	7	6	3.5 T	0.05 × 0.05 × 2	180–240	Iopamidol; acidoCEST	[109]
	2018	14	3	2	0.2 × 0.2 × 1	1140	RARE sequence; WASSR	[110]
	2017	7	6	4	0.469 × 0.312 × 2	~282	Contrast agent: GR- 4Am-SA; catalyCEST	[111]
Liver	2019	11.7	3	2.4	0.39 × 0.39 × 1	N/A	Contrast agent: iodinated liposome	[112]
Prostate	2021	7	5	3	0.3125 × 0.3125 × 1.5	N/A	Denoising; acidoCEST	[113]
	2019	11.7	3	1.8	0.39 × 0.39 × 1	1242	Contrast agent: dextrans; dexCEST	[114]
Kidney	2019	9.4	4	1.6	0.31 × 0.47 × 1	~3000	Respiratory trigger; glucoCEST	[115]
	2018	7	6	3.5 1.0, 1.5, 2.0	0.453 × 0.453 × 2	254 19.421	Contrast agent; respiration-gated acidoCEST	[116]
	2017	7	2	3.0	0.5 × 0.5 × 0.5	310	Contrast agent; acidoCEST	[117]
	2016	3	5	3	0.3125 × 0.3125 × 1.5	276	Contrast agent; acidoCEST	[118]
-	2018	7	3	4	0.625 × 0.625 × 1	462	Contrast agent; catalyCEST	[119]

MS = Magnetic field strength; Tsat = saturation time; AT = Acquisition time.
